# Seismic scenario simulation and ANN-based ground motion model development on the North Tabriz Fault in Northwest Iran

**DOI:** 10.1007/s10950-024-10264-x

**Published:** 2024-11-20

**Authors:** Caglar Temiz, S. M. Sajad Hussaini, Shaghayegh Karimzadeh, Aysegul Askan, Paulo B. Lourenço

**Affiliations:** 1 Departments of Civil Engineering, Middle East Technical University, Ankara, Türkiye; 2 Departments of Civil Engineering and Earthquake Studies, Middle East Technical University, Ankara, Türkiye; 3 Civil Engineering Department, Institute for Sustainability and Innovation in Structural Engineering (ISISE), ARISE, University of Minho, Guimaraes, Portugal

**Keywords:** Stochastic ground motion simulation, Seismic hazard maps, Modified mercalli intensity (MMI) map, North Tabriz Fault (Tabriz Iran), Artificial neural networks (ANN)-based ground motion model (GMM)

## Abstract

Earthquakes pose significant seismic hazards in urban regions, often causing extensive damage to the built environment. In regions lacking robust seismic monitoring networks or sufficient data from historical events, ground motion simulations are crucial for assessing potential earthquake impacts. Yet, validating these simulations is challenging, leading to notable predictive uncertainty. This study aims to simulate four scenario earthquakes with moment magnitudes of 6.8, 7.1, 7.4, and 7.7 in Iran, specifically investigating variations in fault plane rupture and earthquake hypocenter. The North Tabriz Fault (NTF), located within the seismic gap in northwest Iran, is selected as the case study due to the lack of well-recorded ground motions from severe earthquakes, despite historical evidence of large-magnitude events. Simulations are conducted using a stochastic finite-fault ground motion simulation methodology with a dynamic corner frequency. Validation of the simulations is performed by comparing estimated peak ground motions and pseudo-spectral ordinates with existing ground motion models (GMMs), supplemented by inter-period correlation analysis. Simulation results reveal high hazard levels, especially in the northeastern area near the fault plane. Intensity maps in terms of the Modified Mercalli Intensity (MMI) scale underscore the urgency for comprehensive preparedness measures. Finally, a region-specific GMM is developed using Artificial Neural Networks (ANN) to predict peak ground motion parameters with an online platform accessible to end-users.

## Introduction

From ancient times to the present day, earthquakes have remained one of the most devastating natural disasters, leading to significant casualties and the destruction of infrastructures in seismically active areas. However, recent advancements in technology and computational power have improved the ability to assess potential earthquake hazards, enabling the design of safer structures that help mitigate the effects of earthquakes. In regions with well-established seismic networks that are prone to frequent and severe earthquakes, recorded ground motions can be used as inputs for precise risk assessment studies, thereby improving the safety of the built environment. However, in areas with seismic gaps and limited recorded ground motions, particularly from strong earthquakes at short source-to-site distances, the availability of region-specific ground motion data is often insufficient for defining a design scenario (Rezaeian et al. 2024; Yamamoto and Baker 2013). Additionally, the practice of selecting and scaling ground motions can alter the correlation between the modified motions and their original physical conditions (Hancock et al. 2006; Watson-Lamprey and Abrahamson 2006). In such cases, ground motion simulations offer an alternative approach for generating representative time series tailored to the region's seismological features (Arora et al. 2020). These simulations can be used to evaluate the anticipated hazard levels and the resulting potential seismic damage and risk levels in seismic gap regions worldwide.

The literature outlines a range of ground motion simulation techniques, each with varying levels of accuracy and requiring different input-model parameters and computational approaches. These techniques can be broadly categorized into deterministic, stochastic, and hybrid methods (Rezaeian and Sun 2014). Deterministic methods use representative seismic source rupture and velocity models alongside numerical methods to solve the partial differential equations governing wave propagation (Frankel 1993). These methods are most effective for simulating low-frequency contents in regions lacking high-resolution earth models due to the minimum wavelength constraints. In contrast, stochastic methods are better suited for simulating higher-frequency content that is random and incoherent. In the Stochastic method, a far-field S-wave spectrum is enhanced by random phases to generate a mean horizontal ground motion component (David M. Boore 1983). Hybrid methods combine both deterministic and stochastic approaches to accurately simulate the full frequency spectrum and leverage their benefits (Kamae et al. 1998).

Ground motion simulations provide valuable insights into the potential impacts of earthquakes, particularly in regions with limited recorded seismic activity, enabling more accurate risk assessments and the development of targeted mitigation strategies. Ground motion simulations have been widely used in various studies to replicate historical events, evaluate different earthquake scenarios, assess structural demands across various types of structures, and develop ground motion models (GMMs).

Within this context, Karimzadeh and Askan (2018) simulated the historical 1939 Erzincan earthquake in Türkiye, comparing their results to empirical intensity maps. Similarly, Tanırcan and Yelkenci-Necmioğlu (2020) simulated the 2017 Bodrum-Kos earthquake, validating their results through comparison with empirical GMMs and generating seismic hazard maps. Later, Can et al. (2021) simulated the 2002 Cay earthquake in Türkiye and validated their findings by comparing goodness-of-fit scores between observed and simulated motions in addition to comparing their results to empirical intensity maps. These studies underscore the reliability of simulations, when properly validated, in generating robust seismic hazard estimates comparable to empirical GMMs and observed intensities.

In addition to historical event modeling, large suites of simulations have contributed to the development of GMMs, supporting the estimation of peak ground acceleration (PGA) and spectral ordinates (Karimzadeh et al. 2023a; Karimzadeh et al. 2023b; Withers et al. 2020). Simulations have also been used to estimate seismic demands for different structural systems, ranging from single-degree-of-freedom systems (Atkinson et al. 2011) to more complex reinforced concrete and steel structures (Ozsarac et al. 2021; Tsioulou et al. 2019; Xu and Feng 2018).

Building on these developments, Smerzini et al. (2024) developed the BB-SPEEDset, a dataset of near-source, broadband physic-based simulated accelerograms, to bridge knowledge gaps caused by sparse ground motion recordings. They validated the simulations from both seismological and engineering perspectives to ensure reliability for structural assessments. In a related study, Hariri-Ardebili and Rezaeian (2024) explored the applicability of stochastic ground motion simulations for probabilistic seismic performance assessments of geo-structures, such as concrete dams, by developing fragility curves and identifying optimal intensity measures. More recently, Karimzadeh et al. (2024) simulated the 1998 Faial earthquake in the Azores, focusing on the structural demands of masonry buildings, which are often vulnerable to the high-frequency components of seismic motions.

Recent efforts have emphasized the importance of region-specific simulations to enhance pre-earthquake risk assessments, especially in seismically active areas. For instance, Arslan Kelam et al. (2022) and Askan et al. (2024) applied ground motion simulations to evaluate seismic risks in vulnerable regions, emphasizing the importance of scenario modeling for hazard identification and mitigation strategies. The significance of such proactive assessments was highlighted by the February 2023 earthquakes in Kahramanmaras (M_w_ = 7.8) and Elbistan (M_w_ = 7.5) in Türkiye, which resulted in devastating casualties, underscoring the need to evaluate seismic risks before major events occur.

Collectively, these studies showcase the growing importance of ground motion simulations in seismic hazard and risk assessments, as well as in analyzing seismic demands for different structural systems, assuming proper calibration. However, the precision of these assessments largely depends on the selected simulation methodologies and the quality of their calibration and validation processes.

This study aims to simulate ground motions in an important seismic gap located on the North Tabriz Fault (NTF) in Northwest Iran while considering the uncertainty related to rupture effects. Tabriz is selected as the study area due to its designation as one of the world's hazardous regions with a seismic gap. The NTF, characterized by a sharp trace with a North-West to South-East trend, traverses the northern part of the city center. Ghasemi et al. (2021) have examined the anticipated levels of damage resulting from the rupture of two scenario events with moment magnitudes (M_w_) of 7.4 and 7.3. Previous studies have suggested that the NTF has the potential to experience an earthquake with a maximum M_w_ of 7.7 (Solaymani Azad et al. 2015). Despite historical earthquakes that have caused significant loss of life and widespread destruction, there is a lack of recorded strong earthquakes in recent history. This highlights the need to evaluate the potential hazard level of forthcoming seismic events. Although several studies have focused on seismic hazard, vulnerability, and risk assessment in the region (Eslami and Taghizadeh-Farahmand 2020; Karimzadeh et al. 2014; Mohammadi et al. 2020a, b), there remains a significant gap in the investigation of large-magnitude earthquake scenario simulations specific to this area and the development of region-specific GMM.

This study particularly addresses a unique problem by investigating the aleatory uncertainty associated with the fault plane rupture and the location of the hypocenter on the NTF. Through ground motion simulation, and validation against various empirical ground motion models, the seismic hazard in the region is also investigated. To estimate the seismic hazard level in the city center, we use the stochastic finite-fault ground motion simulation method with a dynamic corner frequency approach proposed by (Motazedian and Atkinson 2005). To model the most catastrophic earthquake scenarios, simulations are conducted for potential earthquakes with varying magnitudes, including M_w_ = 7.7, 7.4, 7.1, and 6.8. The simulation results are presented and analyzed through both visual and statistical methods. Initially, to validate the simulations, four ground motion models (GMMs) are used, denoted as BA08 (Boore and Atkinson 2008), AC10 (Akkar and Cagnan 2010), ASB14 (Akkar et al. 2014), and KAAH15 (Kale et al. 2015). BA08 is a globally applicable GMM, while AC10 and ASB14 focus on the Middle Eastern and European regions, and KAAH15 specifically involves data from Eastern Türkiye and Iran. For further validations of the simulated dataset, this study delves into the inter-period correlation of the residuals of pseudo-spectral ordinates. Following the validation steps, anticipated hazard measures are presented using deterministic seismic hazard maps that display various scenario events and their associated uncertainties. Additionally, a mean intensity map based on the Modified Mercalli Intensity (MMI) scale is generated. Finally, a regional artificial neural network (ANN)-based GMM is developed to predict peak ground motion parameters for anticipated scenarios in the region. Ultimately, the study produces an extensive compilation of ground motion time series along with the ANN-based GMM intended for future applications in engineering practice in an online platform.

## Study area

Tabriz is a prominent city in Iran with a rich historical heritage. The archaeological excavations indicate a history of urbanization spanning approximately 5000 years. Presently, Tabriz serves as the capital of East-Azerbaijan Province and is situated in the northwest region of Iran. Geographically, Tabriz lies between eastern Anatolia, the western Caspian Sea, the southern Caucasus thrust belt, and the northern Zagros Mountains range. According to the Statistical Centre of Iran (2020), the population of Tabriz slightly exceeds 3 million. The NTF is a prominent geological feature in the region, consisting of northwestern and southeastern segments that stretch from Mishu Mountain in the west to Bostanabad in the east (Farahani 2018). This fault has an average strike of 115°, a length of approximately 240 km, a dip of predominantly vertical, and a minimum slip rate of 2 mm/year (Karakhanian et al. 2004).

The urban area of Tabriz comprises young and unconsolidated deposits from rivers and glacial sediments of Cenozoic and Quaternary formations. Additionally, Tabriz is located in an alluvial basin (Azarafza and Ghazifard 2016). The alluvial thickness in Tabriz varies by region. In southern Tabriz, it reaches up to 250–300 m. However, in the inhabited areas, including the city center (the actual study area), the alluvial thickness changes from 50 to 100 m (Karimzadeh et al. 2014). Moreover, excavations in the area have revealed the presence of conglomerate, sandstone, and marl layers beneath these alluvial sediments (Azarafza and Ghazifard 2016). The urbanized area is mostly on clay and silt, and marl and sandstone layers (Karimzadeh et al. 2014). The southern and eastern parts of the city are characterized by conglomerate and marl layers, while sandstone formations are found in the northern and northeastern mountainous areas.

**Fig. 1:**
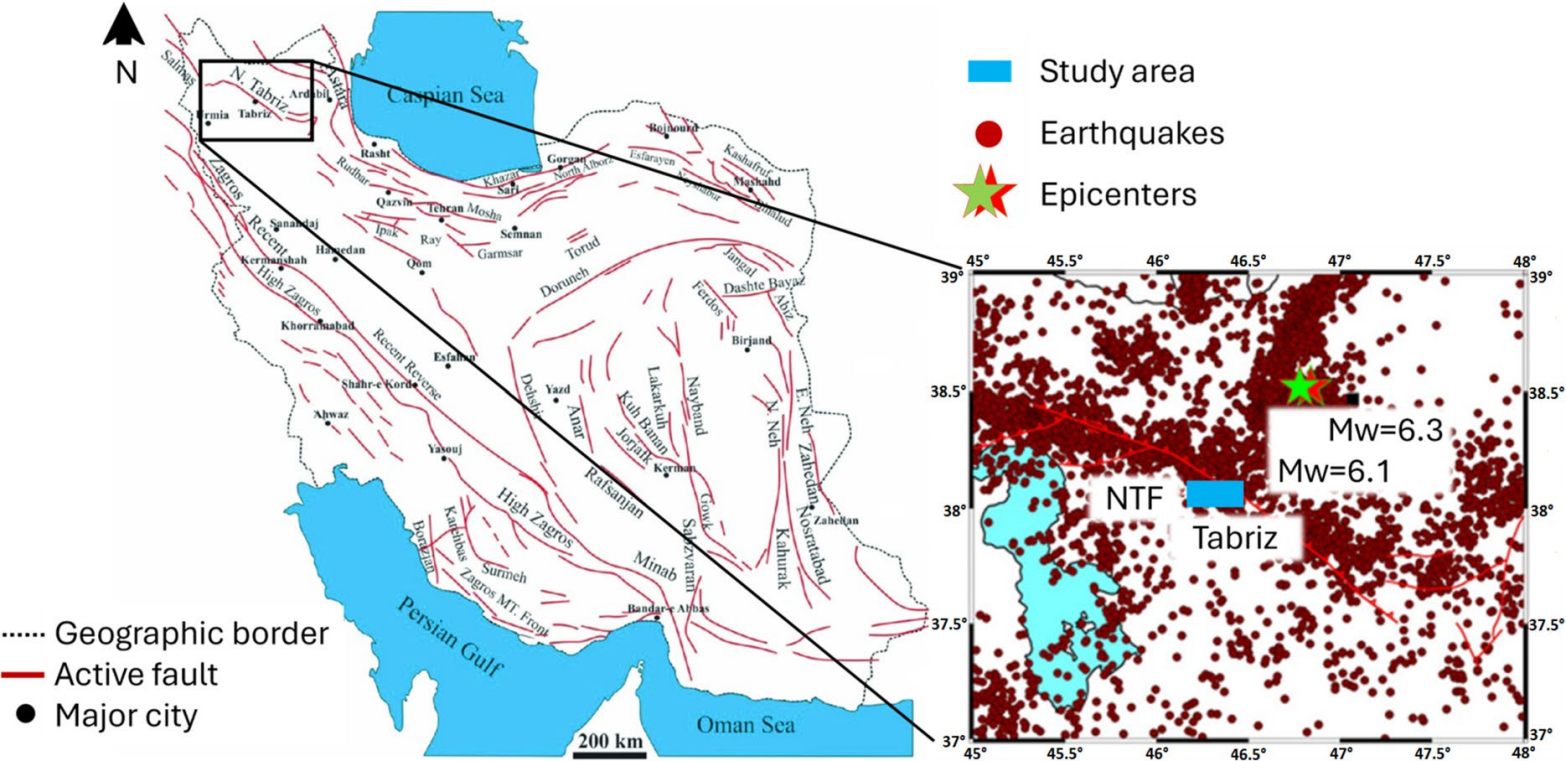
Epicenter of earthquakes within a 20 km radius around the North Tabriz Fault (NTF) (adapted from (Farahani 2018) and (Ghorbani 2021))

Berberian and Arshadi (1976) have investigated the seismic activities of the NTF, including both historical events and those in the twentieth century. Previous studies indicate that Tabriz suffered significant devastation from multiple earthquakes between 634 A.D. and the late 1800s. Notably, three major earthquakes in 1042 (Mw = 7.3), 1721 (Mw = 7.3), and 1780 (Mw = 7.4) caused severe damage through co-seismic surface faulting, as documented in reports from the nineteenth century and earlier (Berberian and Yeats 1999; Hessami et al. 2003). However, the existing descriptions of these events lack sufficient detail to accurately assess the extent of destruction and ground deformations (Berberian and Arshadi 1976). Notably, Tabriz City experienced substantial impacts from the ruptures along the northwestern segment of the fault in 1721 and 1780, as documented by K. Hessami et al. (2009). While seismic activity records became more reliable in the twentieth century, these events did not result in major damage to the city. Figure 1 presents the regional tectonic map with the associated earthquakes.

Given the long interval since the last large-magnitude earthquake (M_w_ ≥ 7.5) and the ongoing activity of the NTF, the potential for future strong earthquakes in the area should not be ignored. Therefore, the study area is centered on Tabriz city center. Kheirizadeh Arouq et al. (2020) conducted a study based on catastrophe theory, revealing that over a quarter (35%) of urban areas are classified as highly vulnerable to seismic activity. Additionally, Tabriz is identified as a very highly susceptible region on the earthquake susceptibility map developed by Mohammadi et al. (2020a)

**Fig. 2:**
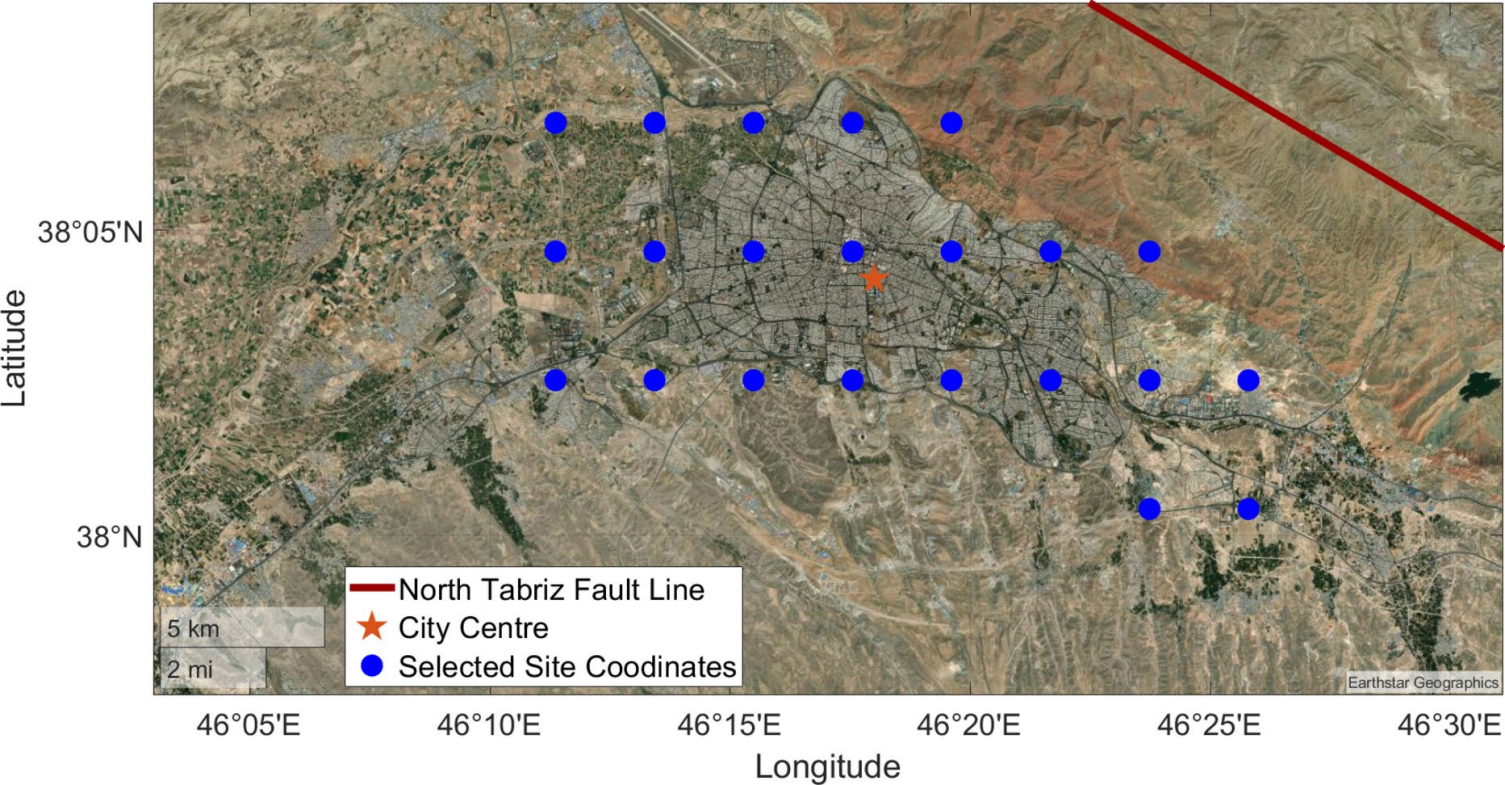
Fault line and selected stations for ground motion simulations

This study simulates potential strong earthquakes and obtains simulated ground motions from 22 designated dummy stations within Tabriz city center, as illustrated in Fig. 2. These stations are selected based on high levels of urbanization and human population. The NTF trace is shown with the red line, while the blue dots indicate the location of dummy stations within the city center. These dummy stations are positioned in areas with important structures such as residential buildings, airports, hospitals, and other essential facilities.

## Ground motion simulation methodology

In regions lacking well-studied local source and velocity models, the stochastic methodology is considered effective and preferred for simulating ground motions. This method is particularly advantageous for capturing the aleatory uncertainty associated with earthquakes, thereby accounting for the inherent variability in ground motions. However, it primarily focuses on simulating the random intermediate and medium- to high-frequency components of ground motions (Beresnev and Atkinson 1997; David M. Boore 1983a, 1983b; Motazedian and Atkinson 2005). While this is suitable for many engineering applications, such as for structures sensitive to these frequency ranges, the approach does not fully capture low-frequency components. Low-frequency ground motions, which are important for the response of tall buildings and other long-period structures, require more complex deterministic physics-based simulations or a hybrid simulation scheme. Despite these limitations, stochastic techniques are known to accurately simulate ground motion amplitudes within the frequency range of primary interest to engineers (Boore 2009; Karimzadeh 2019; VahidiFard et al. 2017).

For the simulation of the scenario events in this study, the stochastic finite-fault ground motion simulation technique developed by (Motazedian and Atkinson 2005) is employed using the EXSIM platform. This approach represents the fault plane using a rectangular geometry and models it as a collection of smaller sub-faults that act as point sources (David M Boore 1983a, 1983b). The acceleration response spectrum in the frequency domain is modeled for each point source, taking into account the contributions of source, path, and site effects. Subsequently, the responses for all sub-faults are summed in the time domain to obtain the final response of the main fault as follows:

1 $$\mathrm{a}\left(\mathrm{t}\right)= {\sum }_{\mathrm{i}=1}^{\mathrm{nl}}\sum_{\mathrm{j}=1}^{\mathrm{nw}}{\mathrm{a}}_{\mathrm{ij}}(\mathrm{t}+ {\Delta \mathrm{t}}_{\mathrm{ij}}),$$

where $\mathrm{a}\left(\mathrm{t}\right)$ is the total seismic signal at a time t, nl and nw are the number of sub-faults along the length and width of the main fault, respectively, and ${\Delta \mathrm{t}}_{\mathrm{ij}}$ is the relative delay time for the radiated wave from the *ij*th sub-fault to reach the observation point. For each sub-fault (David M Boore 1983a, 1983b), the acceleration spectrum is computed as:

2 $${A}_{ij}\left(f\right)={CM}_{{0}_{ij}}{H}_{ij}\left[{\left(2\pi f\right)}^{2}/\left[1+{\left(\frac{f}{{f}_{{c}_{ij}}}\right)}^{2}\right]\right]{e}^{-\frac{\pi f{R}_{ij}}{Q\left(f\right)\beta }}G\left({R}_{ij}\right)A\left(f\right){e}^{-\pi Kf}$$

Where $C=\frac{{R}^{\theta \varphi }FV}{4\pi \rho {\beta }^{2}}$ is a constant, with ${R}^{\theta \varphi }$ as the radiation pattern (average value of 0.55 for shear waves, F as free surface amplification (2.0), V as partition onto two horizontal components (0.71), $\uprho$ as the density, and $\upbeta$ as the shear-wave velocity. ${\mathrm{M}}_{{0}_{\mathrm{ij}}}= \frac{{\mathrm{M}}_{0}{\mathrm{S}}_{\mathrm{ij}}}{{\sum }_{\mathrm{k}=1}^{\mathrm{nw}}\sum_{\mathrm{l}=1}^{\mathrm{nl}}{\mathrm{S}}_{\mathrm{kl}}}$ is the seismic moment, ${\mathrm{S}}_{\mathrm{ij}}$ is the relative slip weight and ${\mathrm{f}}_{{\mathrm{c}}_{\mathrm{ij}}}\left(\mathrm{t}\right)$ is the dynamic corner frequency of the ${\mathrm{ij}}^{\mathrm{th}}$ sub-fault where ${\mathrm{f}}_{\mathrm{ij}}\left(\mathrm{t}\right)={{\mathrm{N}}_{\mathrm{R}}\left(\mathrm{t}\right)}^{-1/3}4.9\times {10}^{6}\upbeta {(\frac{\Delta\upsigma }{{\mathrm{M}}_{{0}_{\mathrm{ave}}}})}^{1/3}$. Here, $\Delta\upsigma$ is the stress drop, ${\mathrm{N}}_{\mathrm{R}}\left(\mathrm{t}\right)$ is the cumulative number of ruptured sub-faults at time $\mathrm{t}$, and ${\mathrm{M}}_{{0}_{\mathrm{ave}}}=\frac{{\mathrm{M}}_{0}}{\mathrm{N}}$ is the average seismic moment of subfaults. ${\mathrm{S}}_{\mathrm{ij}}$ is the distance from the observation point, $\mathrm{Q}(\mathrm{f})$ is the quality factor, $\mathrm{G}\left({\mathrm{R}}_{\mathrm{ij}}\right)$ is the geometric spreading factor, $\mathrm{A}(\mathrm{f})$ represents the site amplification term, and ${\mathrm{e}}^{-\uppi \mathcal{K}\mathrm{f}}$ is a high-cut filter included to provide the spectral decay at high frequencies described with the Kappa factor of soils (Anderson and Hough 1984). ${\mathrm{R}}_{\mathrm{ij}}$ is a scaling factor introduced to conserve the high-frequency spectral level of the subfaults. The term ${H}_{ij}=(N*{(\sum ({\mathrm{f}}^{2}/[1+{\left(\frac{\mathrm{f}}{{\mathrm{f}}_{0}}\right)}^{2}])/\sum (({\mathrm{f}}^{2}/[1+{\left(\frac{\mathrm{f}}{{\mathrm{f}}_{{0}_{ij}}}\right)}^{2}])))}^{1/2}$ corresponds to a scaling factor for conversation of the spectral shape at higher frequencies.

To simulate the scenario events for each magnitude, the length and width of the ruptured fault are estimated using the study by Wells and Coppersmith (1994). The entire length of the NTF is approximately 240 km (Farahani 2018; Solaymani Azad et al. 2015). Alternative fault planes are considered to capture the variability of the rupture plane. For the largest event with M_w_ = 7.7, the NTF is divided into 17 alternative ruptured fault lines, each spanning a length of 140 km, to generate various possible scenarios. Similarly, for the other magnitudes, the NTF is divided into more than 13 events for each case. Specifically, for M_w_ = 7.4, the fault plane is divided into 12 segments, each with a length of 84 km; for M_w_ = 7.1, it is divided into 13 segments with a length of 52 km, and for M_w_ = 6.8 it is divided into 15 segments with a length of 32 km. The respective widths of these ruptured fault planes are determined to be 24 km, 20 km, 16 km, and 12 km for M_w_ = 7.7, 7.4, 7.1, and 6.8, respectively. It is noted that for all scenarios with events of the same magnitude, the epicenter is shifted along the fault plane by an equal distance.

In addition to the variability of the ruptured fault plane, the study also investigates the randomness associated with the hypocenter location for alternative scenario events. To achieve this, the focal depth and epicenter location are treated as random variables. Moradi et al. (2011) found that the median focal depth for earthquakes in the Tabriz region is approximately 12.1 km, with a standard deviation of around 4.9 km. In this study, to incorporate the uncertainty related to the focal depth, a range of 6.0 km to 18.0 km is considered. Additionally, the epicenter location is assumed to change along the length of the ruptured fault plane.

Excluding the random parameters discussed above, the other input-model parameters are determined in a deterministic manner across all magnitudes and are essential for completing the simulations. These parameters are sourced from various studies. The strike and dip angle values, derived as 115° and 90°, respectively, are obtained from the studies by Farahani (2018) and Ghayamghamian and Rajool (2012). The quality factor is taken from the study conducted by Jafarian Vernosfaderani et al. (2019), while the kappa factor is derived as 0.035 based on the research conducted by Amiranlou et al. (2016). The duration model follows the work of Herrmann (1985) as applied in Hoveidae et al. (2021) for the simulation of scenario earthquakes in the Tabriz region. This study uses site amplifications from Boore and Joyner (1997) for a generic soil class with a Vs30 value of 310 m/s. This value is selected because the dummy stations are mainly located in areas with Eurocode soil Class C (Code Price 2005), with Vs30 values ranging from 300 to 360 m/s, as indicated by GIS and DEM-based seismic site condition studies by Karimzadeh et al. (2017, 2019). Yaghmaei-Sabegh and Hassani (2020) further corroborate this by reporting an average Vs30 value of approximately 300 m/s for the region, reflecting minimal soil variability based on analyses of seismic events in Iran. Although soil variability is important in improving ground motion simulations, this study adopts a constant Vs30 value to focus on uncertainties related to the fault rupture plane and hypocenter location, which limits the results to a generic soil classification. The stress drop is calculated using the empirical equation given by Mohammadioun and Serva (2001). Geometrical spreading, pulsing percent, rupture velocity, density, and window type are assumed to be the same as in the research conducted by Amiranlou et al. (2016) and Hoveidae et al. (2021). A summary of all this information can be found in Table 1.

**Table 1:** Deterministic input parameters for ground motion simulation of scenario events with different magnitude values

Parameters	Value			
M_w_	7.7	7.4	7.1	6.8
Length (km)	140	84	52	32
Width (km)	24	20	16	12
Sub-fault length (km)	4	4	4	4
Sub-fault width (km)	4	4	4	4
Stress Drop (bars)	110	95	80	65
Fault Mechanism	Strike-slip			
Strike (^o^)	115			
Dip (^o^)	90			
Shear Wave Velocity (km/s)	3.3			
Rupture Velocity/Shear Wave Velocity	0.8			
Density (g/cm^3^)	2.8			
Pulsing Percent	35			
Quality Factor	$103{\mathrm{f}}^{0.88}$			
Geometrical Spreading	${\mathrm{R }}^{-1}\mathrm{ R}\le 85$ ${\mathrm{R }}^{-0.5}\mathrm{ R}>85$			
Duration model	T = T_0_ + 0.05 (T_0_: Source duration)			
Kappa Value	0.035			
Window Type	Saragoni-Hart			
Site Amplifications	Generic soil			
Sampling Time (s)	0.005			

## Simulated ground motion dataset

**Fig. 3:**
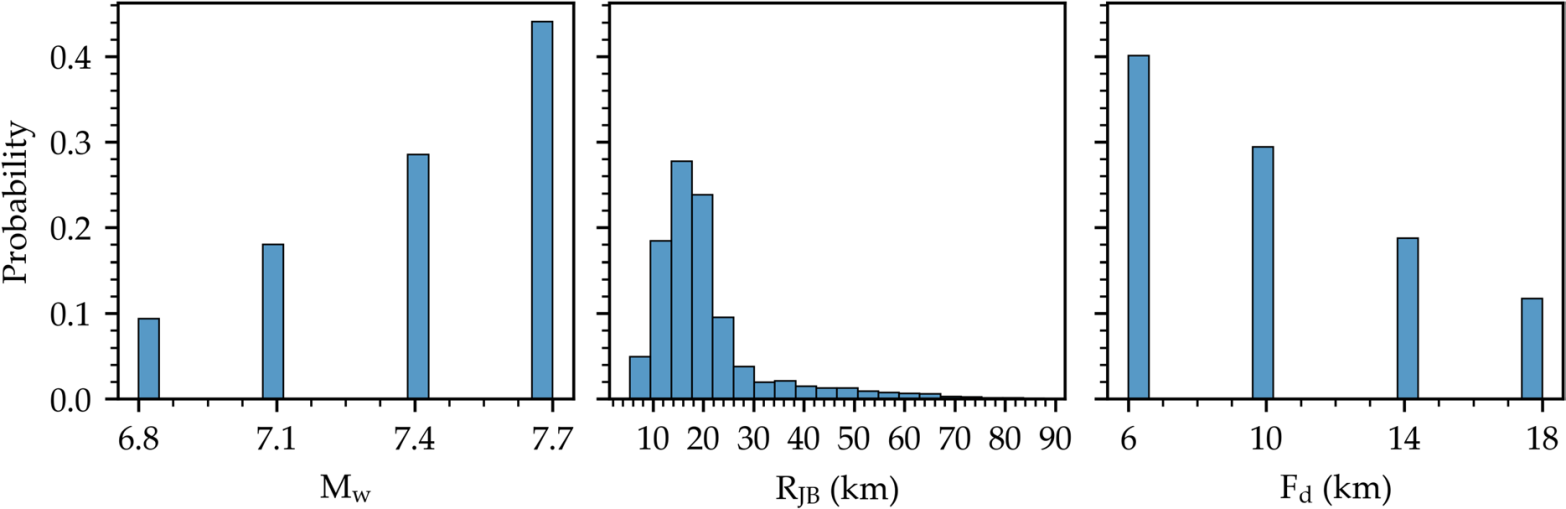
Histograms of seismological parameters of the simulated dataset

**Fig. 4:**
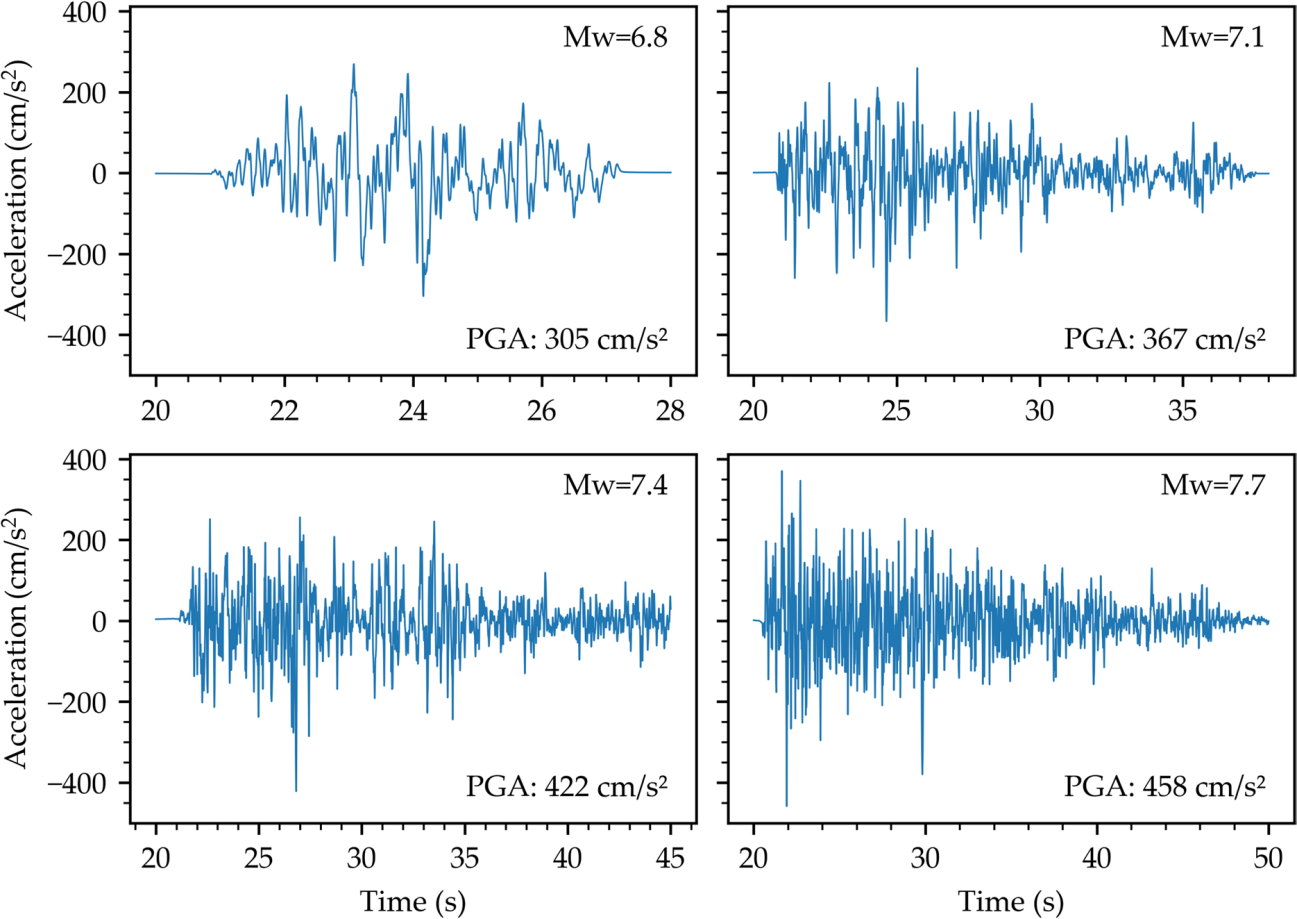
Samples of simulated time series for the study area

A total of 206,382 simulated ground motion time series have been generated for Tabriz City at the selected stations. The dataset encompasses earthquake scenarios with M_w_ of 6.8, 7.1, 7.4, and 7.7, and Joyner-Boore distances (R_JB_) ranging from 5.4 km to 87 km with focal depths (F_d_) between 6 and 18 km. Given that soil class C predominates in the region, we used a representative Vs30 value of 310 m/s under a strike-slip fault mechanism. The seismological parameters of the dataset are illustrated in Fig. 3. It is noteworthy that a higher number of simulations have been generated for larger M_w_ values, shorter R_JB_ distances, and shallower focal depths to address the scarcity of strong near-fault recordings. Figure 4 presents sample simulated ground motions for scenario events with M_w_ of 7.7, 7.4, 7.1, and 6.8, at a R_JB_ distance of 12 km, and a F_d_ of 10 km along with their respective peak ground acceleration (PGA) values.

## Assessment of simulations

This section focuses on validating the simulations through comparison with GMMs, analyzing attenuation of the simulated data, and developing seismic hazard and MMI maps.

### Validation of simulations

It is important to validate the simulated motions before they can be used for engineering applications. Validation methods vary depending on the availability of recorded motions. In cases where recorded motions are absent, a commonly employed approach involves using empirical GMMs for validation purposes. The literature offers numerous parametric and non-parametric GMMs. However, selecting appropriate GMMs for comparison is crucial, especially those that exhibit similar seismological and tectonic characteristics to the region under study. In this study, four distinct GMMs are used for the validation process, namely BA08 (Boore and Atkinson 2008), AC10 (Akkar and Cagnan 2010), ASB14 (Akkar et al. 2014), and KAAH15 (Kale et al. 2015). These four selected GMMs fulfill different purposes. The BA08 model is recognized as a global GMM due to its development, incorporating recorded data from diverse global locations. In contrast, the AC10 model serves as a local model, specifically tailored to the seismic characteristics of Türkiye. The ASB14 model is a regional one developed to capture the seismic attributes of the Middle Eastern and European regions, encompassing multiple countries. Lastly, the KAAH15 model falls under the category of a local GMM, customized to account for the seismic behavior observed in Iran and Türkiye. The validation primarily focuses on five essential intensity measures (IMs): PGA, Peak Ground Velocity (PGV), and Pseudo-Spectral Acceleration (PSA) with a 5% damping ratio for periods of T = 0.2 s, 0.5 s, and 1.0 s. These parameters serve as representative indicators for evaluating the accuracy and reliability of the simulated dataset.

**Fig. 5:**
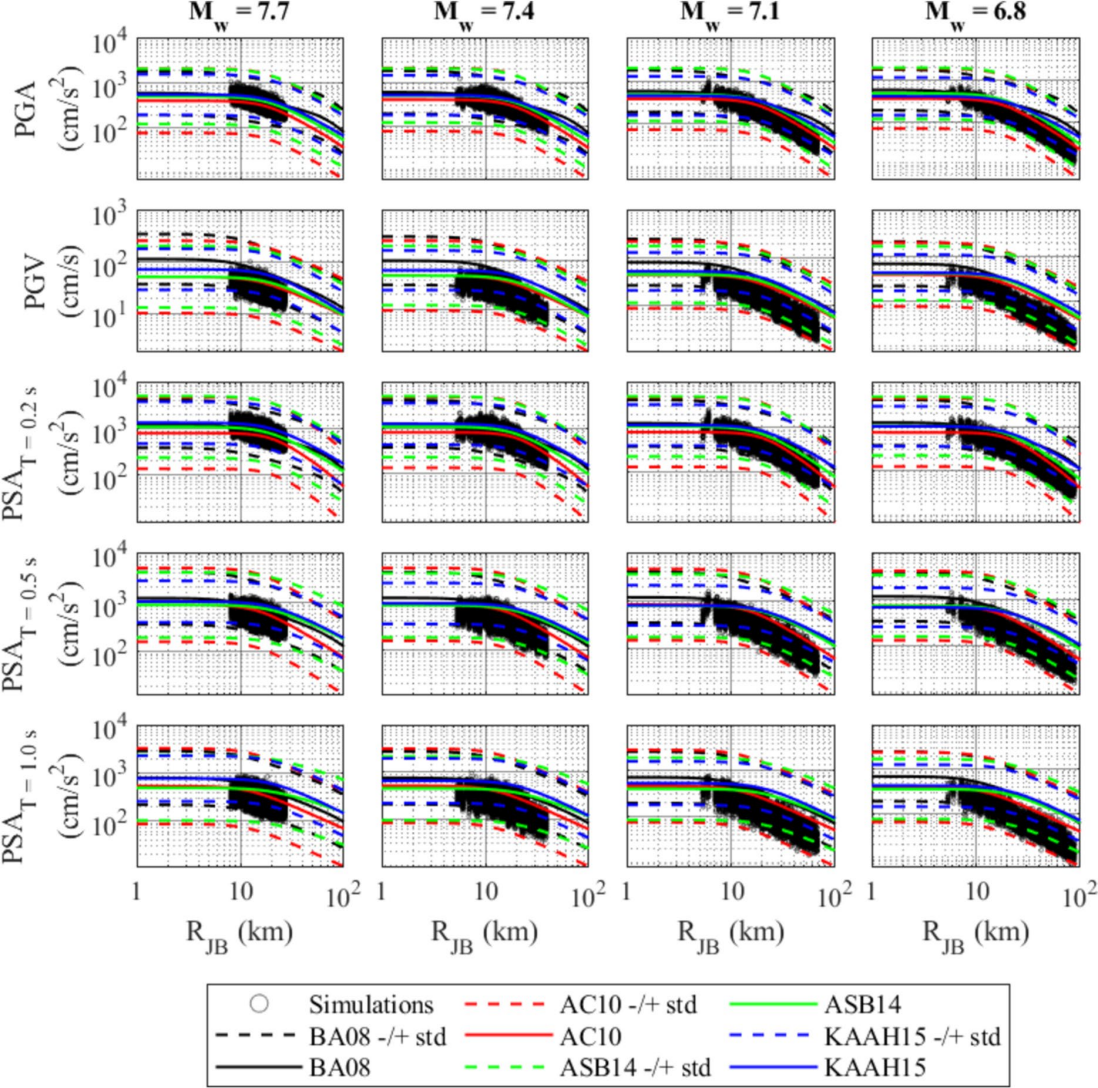
Comparison of the simulated ground motion IMs, namely PGA, PGV, and PSA at T = 0.2 s, 0.5 s, and 1.0 s against selected GMMs for the scenario events of M_w_ = 7.7, 7.4, 7.1, and 6.8

Figure 5 illustrates the distance attenuation of simulated ground motion IMs (i.e., PGA, PGV, and PSA at T = 0.2 s, 0.5 s, and 1.0 s) in comparison to estimates from the GMMs. The results indicate that the simulated IMs generally fall within ± 2 standard deviations (std) of the GMM estimates. For distances less than 20 km, the simulated intensity levels closely align with all GMMs. However, the attenuation patterns for simulated IMs are noticeably faster at distances exceeding 20 km, particularly for longer-period PSA. These discrepancies can be attributed to various parameter settings in the finite fault stochastic simulation method. Key factors, such as fault geometry (length, width, and depth), stress drop, and rupture velocity, influence how seismic energy is released and propagated. The choice of geometrical spreading model significantly affects how IMs decay with distance, while the quality factor and kappa value represent energy dissipation in the medium and site effects, respectively. Overall, these parameter settings contribute to the observed differences between simulated IMs and those from empirical GMMs, underscoring the importance of conducting region-specific studies to refine these parameters. The present research attempts to assess not only the attenuation of the representative IMs but also to determine the anticipated seismic hazard level within the study area.

**Fig. 6:**
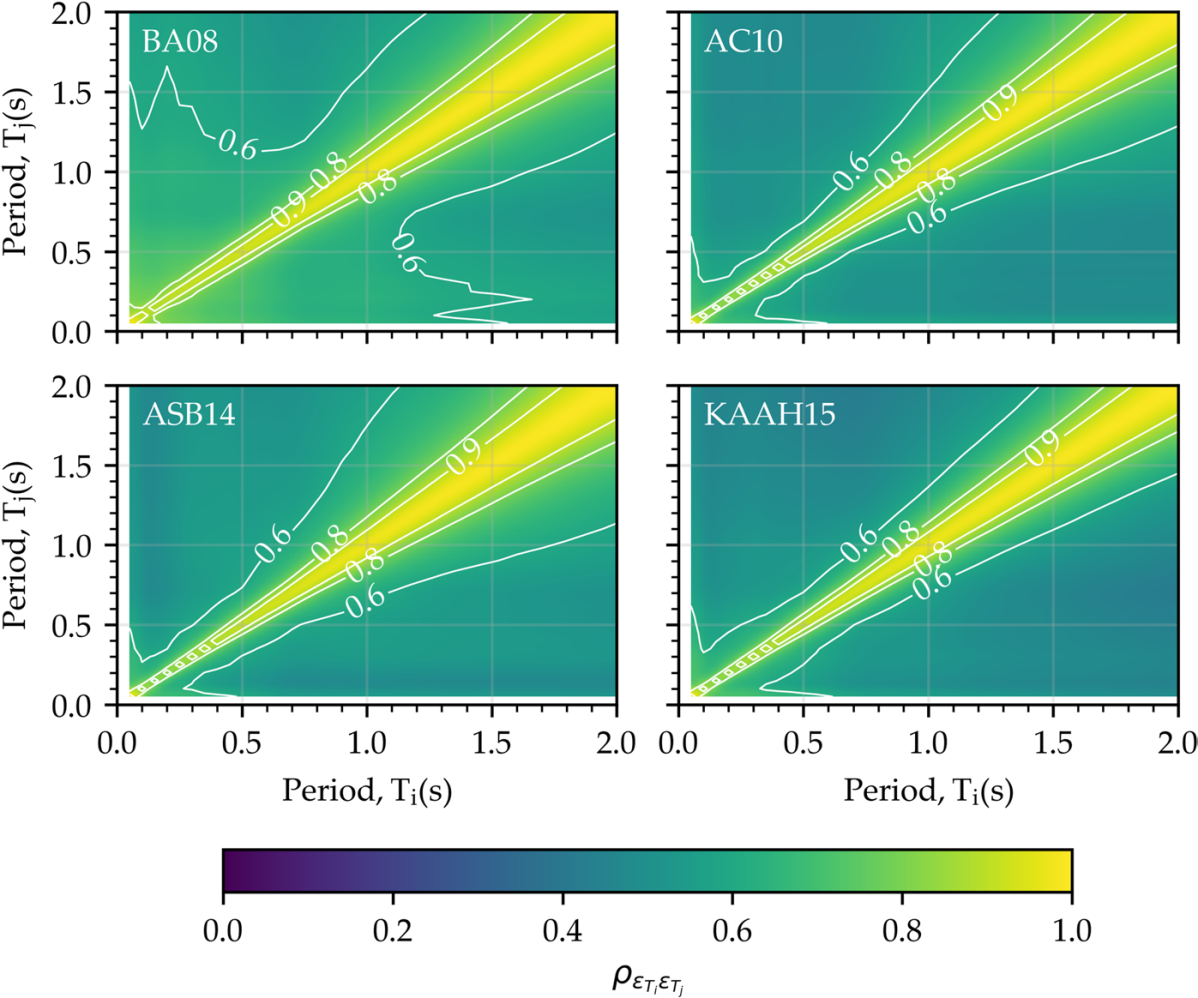
${\rho }_{\epsilon }$ of PSA contours over a period of 0.05–2 s regarding each selected GMM

As a further step in the validation process, the correlations between normalized residual ($\upepsilon$) of PSA values for various vibration periods are investigated. Recently, Altindal and Askan (2023) validated a simulated ground motion dataset generated for the entire Türkiye with this approach yielding a robust assessment. Given a PSA from an earthquake scenario with magnitude M_w_ at a recorded distance R_JB_, $\upepsilon$ value can be calculated as (Baker 2011):

3 $$\upepsilon (\mathrm{T})=\frac{\mathrm{lnPSA}\left(\mathrm{T}\right)-{\upmu }_{\mathrm{lnPSA}}({\mathrm{M}}_{\mathrm{w}}, {\mathrm{R}}_{\mathrm{JB}},\mathrm{ T})}{{\upsigma }_{\mathrm{lnPSA}}(\mathrm{T})}$$

where ${\upmu }_{\mathrm{lnPSA}}({\mathrm{M}}_{\mathrm{w}}, {\mathrm{R}}_{\mathrm{JB}},\mathrm{ T})$ and ${\upsigma }_{\mathrm{lnPSA}}(\mathrm{T})$ are the mean value and standard deviation of lnPSA(T) estimated from GMM, respectively. lnPSA(T) is the natural logarithm of observed PSA values, referred to herein as the simulation. In this study, the $\upepsilon$ values are calculated for all the simulated motions in the dataset for a period range of 0.05 s to 2 s, regarding each of the selected GMMs. In seismic demand assessment of multi-degree-of-freedom (MDOF) systems that are subjected to various periods of excitation, the correlation coefficient of $\upepsilon$ across the period range (${\uprho }_{\upepsilon }$) is a crucial indicator (Burks and Baker 2014). Therefore, ${\uprho }_{\upepsilon }$ of PSA, obtained using Pearson product-moment correlation coefficient, regarding each selected GMM is illustrated in Fig. 6 over the considered period range. These contours indicate that the correlations calculated based on BA08 exhibit the highest inter-period correlation, whereas those derived from AC10 and KAAH15 yield the lowest correlations. However, ${\uprho }_{\upepsilon }$ values regarding ASB14 exhibit high inter-period correlations towards longer periods. Nonetheless, a consistent pattern is observed across all GMMs for both inter-period correlations and attenuations of considered intensity measures, affirming the validity of the simulated dataset.

### Seismic hazard and MMI maps

**Fig. 7:**
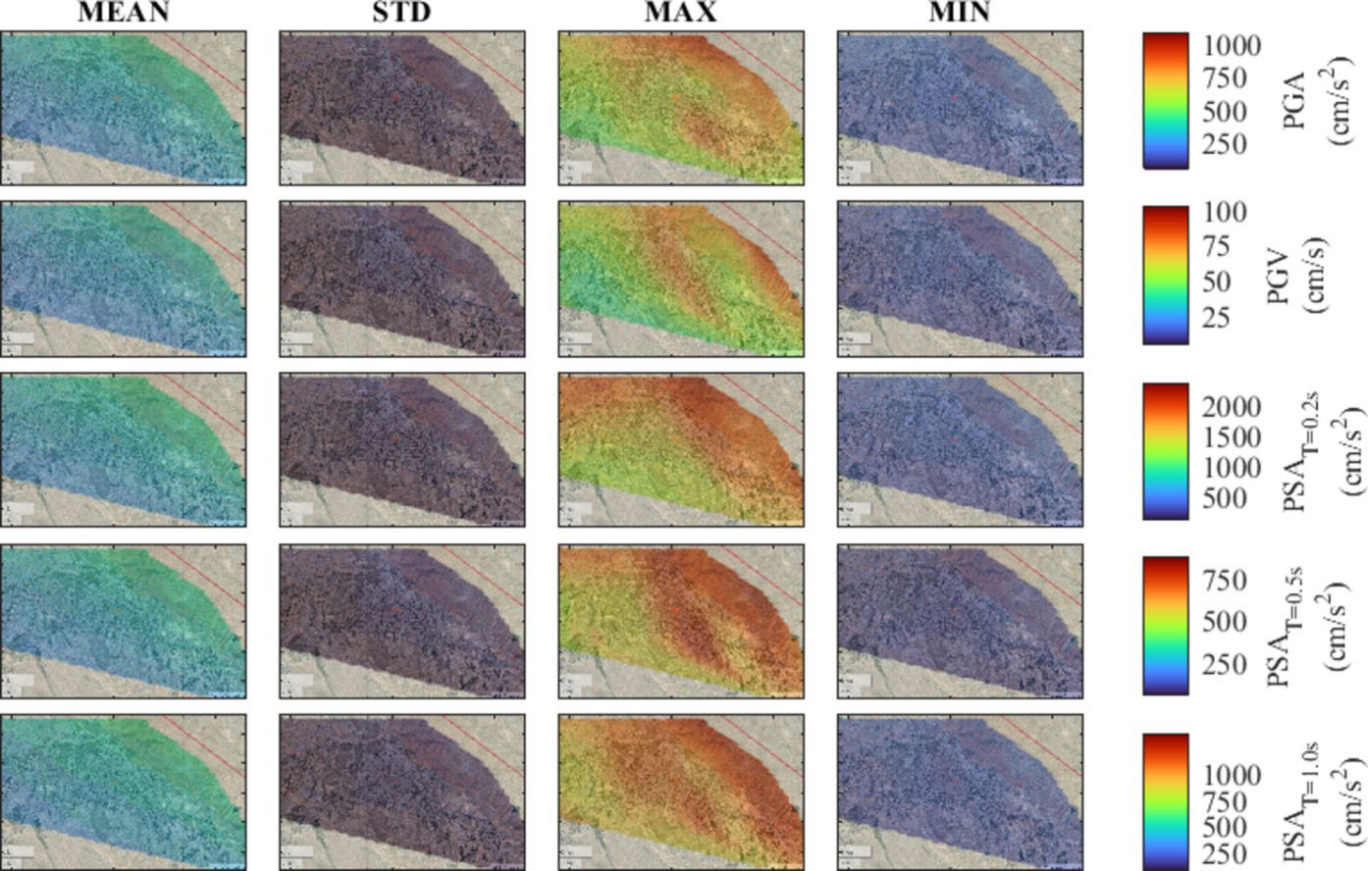
Seismic hazard maps for M_w_ = 7.7, in terms of PGA, PGV, PSA at T = 0.2 s, 0.5 s, and 1.0 s

**Fig. 8:**
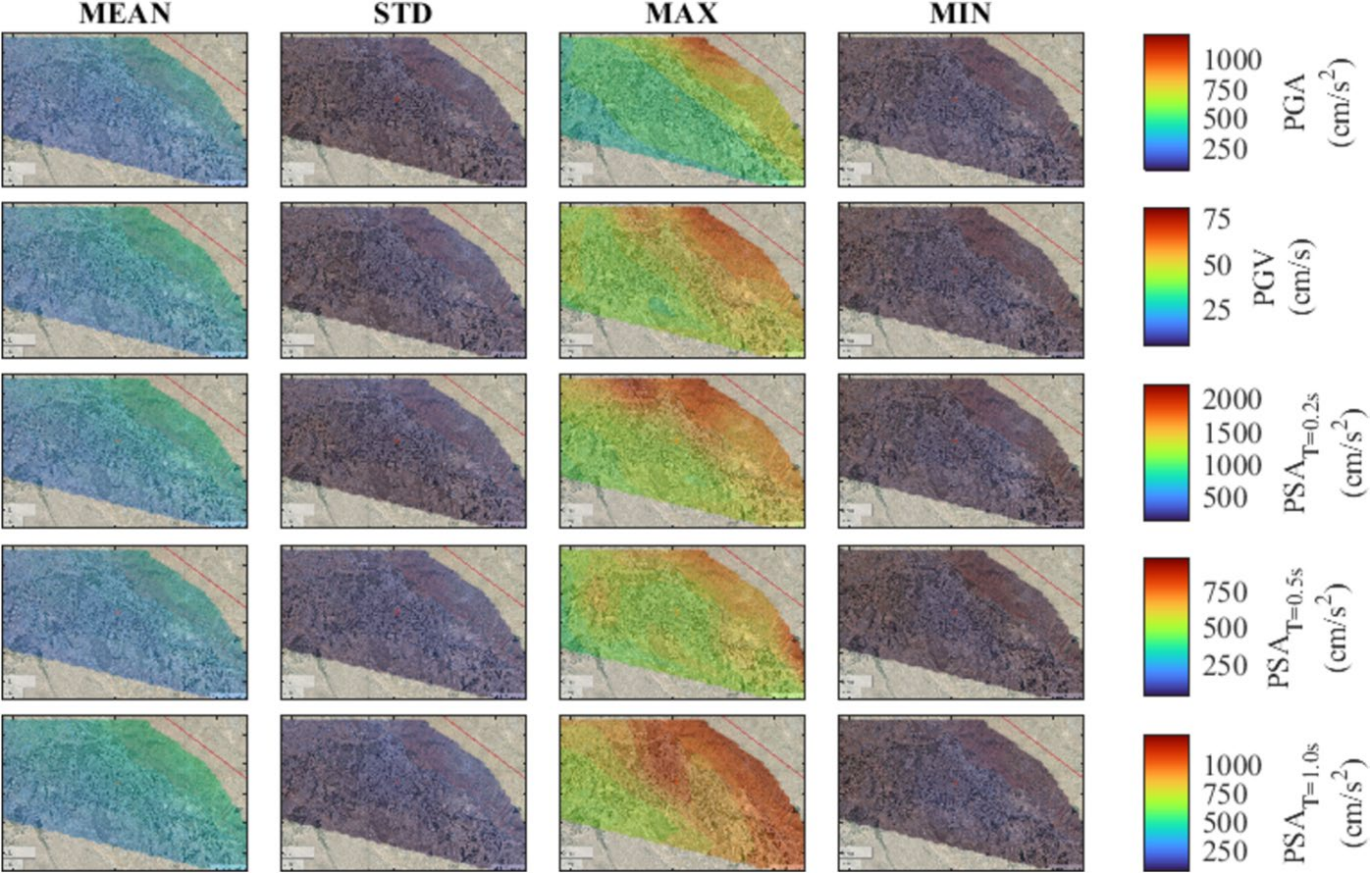
Seismic hazard maps for M_w_ = 7.4, in terms of PGA, PGV, PSA at T = 0.2 s, 0.5 s, and 1.0 s

**Fig. 9:**
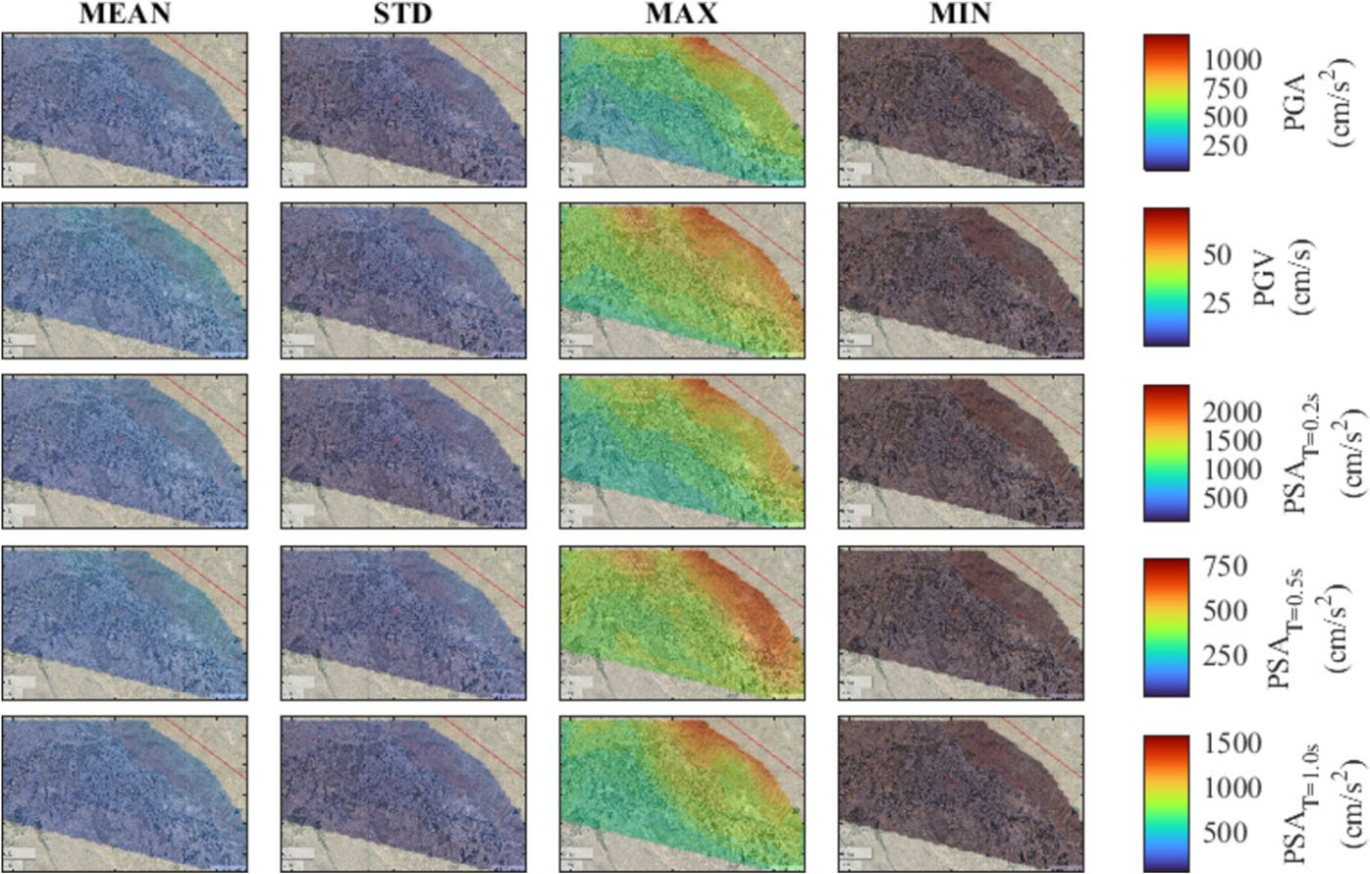
Seismic hazard maps for M_w_ = 7.1, in terms of PGA, PGV, PSA at T = 0.2 s, 0.5 s, and 1.0 s

**Fig. 10:**
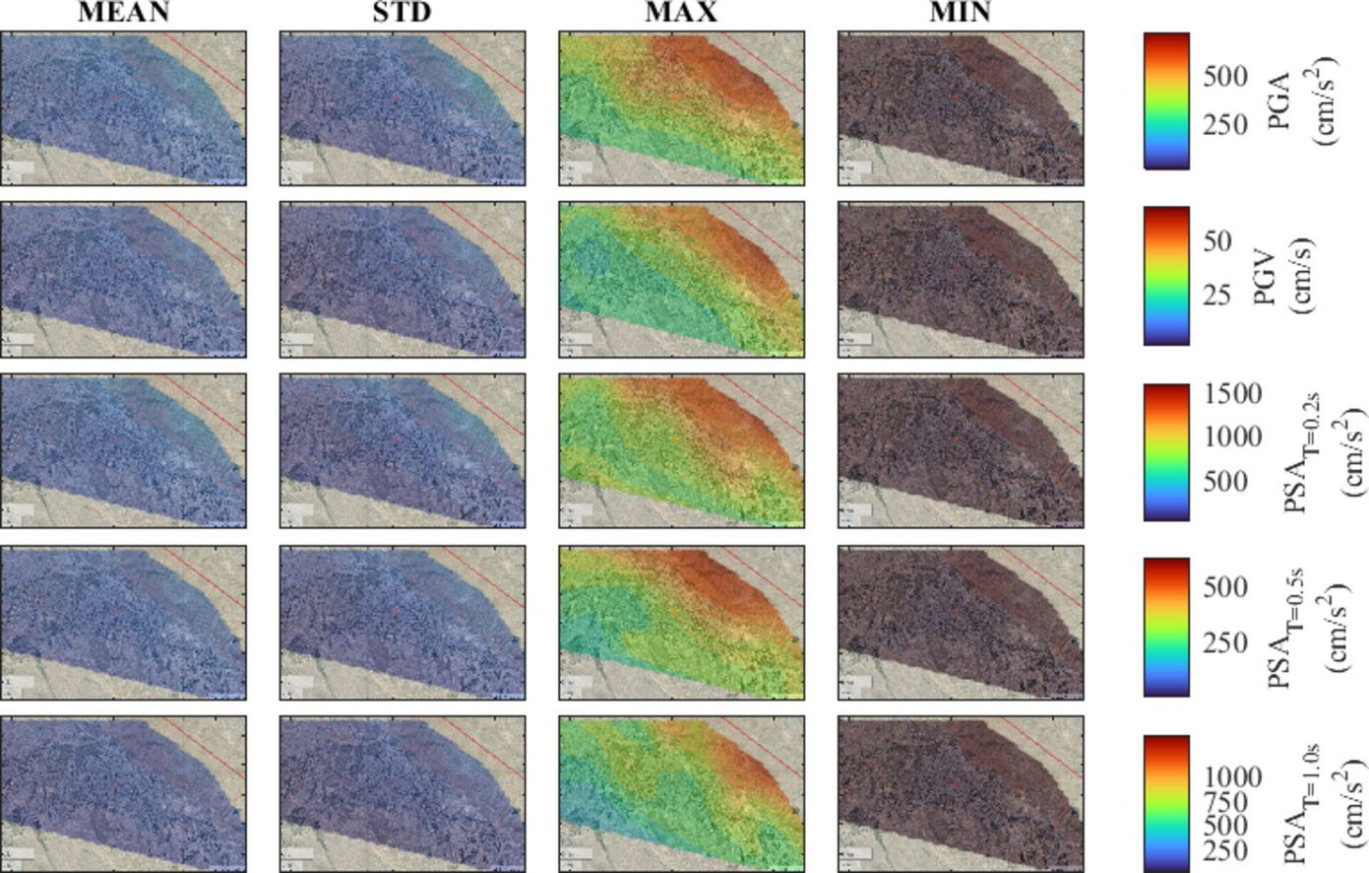
Seismic hazard maps for M_w_ = 6.8, in terms of PGA, PGV, PSA at T = 0.2 s, 0.5 s, and 1.0 s

To provide a comprehensive understanding of ground motion intensity levels for different scenario events, seismic hazard maps are developed for the region. To study the influence of various hypocenter locations and fault plane rupture configurations associated with each magnitude, the mean and standard deviation along with the minimum and maximum values of considered IMs are computed. The computations are conducted for M_w_ of 7.7, 7.4, 7.1, and 6.8 for the city center, allowing for the assessment of the IMs variability. The results are illustrated in Figs. 7, 8, 9, and 10, corresponding to decreasing M_w_ values. Notably, as the magnitude increases from 6.8 to 7.7, there is a consistent trend of increasing mean values for PGA, PGV, and PSA at all periods. In particular, comparing the mean value of the IMs for M_w_ of 7.7 with those of M_w_ = 6.8, 7.1, and 7.4, indicates an overall average decline of approximately 65%, 45%, and 15%, respectively.

Overall, the spatial variability of ground motion parameters in Tabriz highlights the presence of inherent uncertainties arising from variations in hypocenter location and fault plane rupture. As expected, the findings indicate that sites closer to the fault line exhibit increased ground motion intensities. This observation implies that under the assumption of uniform soil conditions, the near-fault locations show the most variability with increased ground motion levels. The identification of such trends emphasizes the importance of accounting for the stochastic nature of input parameters in simulations for future studies. Finally, the analysis indicates that in the earthquake scenarios being considered, the city center can face an increased level of hazard, emphasizing the urgent need for attention to mitigate seismic risk.

To better understand projected intensity levels, MMI maps are generated for all scenario events based on both PGA and PGV. Two regression models are employed to estimate MMI, using PGA and PGV as representative IMs to assess potential damage to both rigid and semi-flexible structures, respectively. To this end, empirical conversion equations between felt intensity and recorded ground motion IMs proposed by Bilal and Askan (2014) are used:

4 $$\mathrm{MMI}=0.132+3.884\times \mathrm{log}(\mathrm{PGA})$$

5 $$\mathrm{MMI}=2.673+4.340\times \mathrm{log}(\mathrm{PGV})$$

**Fig. 11:**
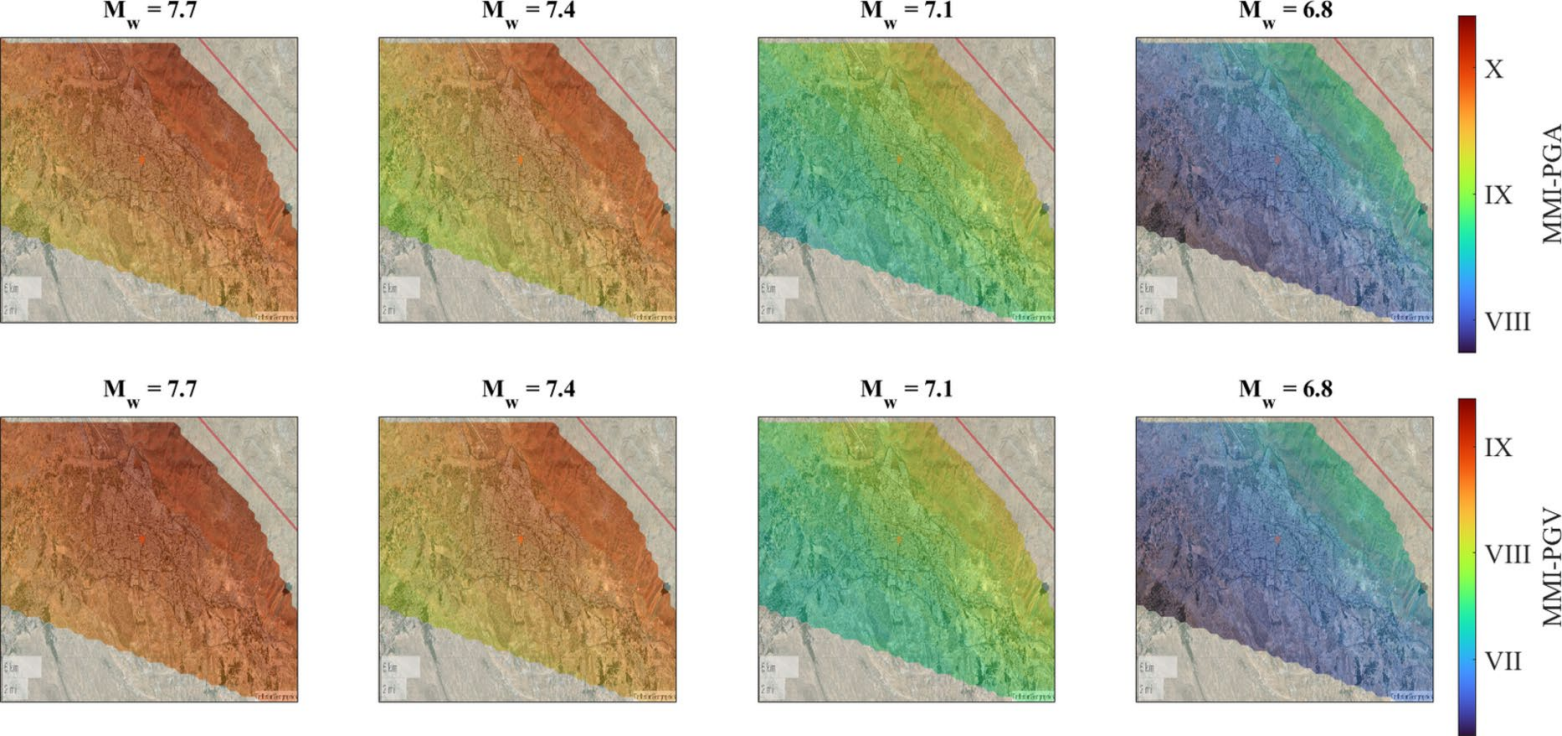
Mean MMI maps obtained using MMI-PGA and MMI-PGV relationship for M_w_ = 7.7, 7.4, 7.1, and 6.8

Figure 11 illustrates the MMI maps in terms of PGA and PGV, respectively. The PGA-based map is useful for representing the behavior of masonry structures while the PGV-based map better depicts the flexible nature of the reinforced concrete structures in the region. The city center is expected to experience a mean MMI level ranging from VII to X depending on the magnitude. Specifically, M_w_ of 7.7 and 7.4 scenarios indicate a state of severe damage. It is noteworthy that MMI values are derived from mean PGA and PGV values, implying that the inherent uncertainty could amplify the expected damage level. This conclusion emphasizes the critical need for pre-earthquake risk assessment studies, underscored by the recent Türkiye earthquake (February 2023).

## Ground motion modeling methodology

Ground motion models represent a widely used method for estimating ground motion IMs, such as PGA, PGV, or PSA. Typically, these models are developed using statistical regression analysis on large datasets of ground motion IMs (e.g., (Akkar et al. 2014; Akkar and Cagnan 2010; Boore and Atkinson 2008; Kale et al. 2015)). GMMs are generally expressed as:

6 $$\mathrm{ln}\left({\mathrm{y}}_{\mathrm{ij}}\right)=\left(\begin{array}{c}ln(PGA)\\ \mathrm{ln}\left(\mathrm{PGV}\right)\\ \mathrm{ln}\left({\mathrm{PSA}}_{0.05\mathrm{s}}\right)\\ .\\ .\\ .\\ \mathrm{ln}\left({\mathrm{PSA}}_{2\mathrm{s}}\right)\end{array}\right) =\mathrm{f}\left({\mathrm{M}}_{\mathrm{w}}, {\mathrm{R}}_{\mathrm{jb}}, {\mathrm{F}}_{\mathrm{d}},\dots \right)+{\upeta }_{\mathrm{i}}+{\upepsilon }_{\mathrm{ij}}$$

where $\mathrm{ln}\left({\mathrm{y}}_{\mathrm{ij}}\right)$ represents the natural logarithm of the considered IMs (e.g., PGA, PGV, and PSA). f(M_w_, R_JB,_ F_d_) is the median of the model, R_JB_ is the Joyner-Boore distance and F_d_ is the focal depth. Within this notation, the between-event and within-event residual components are denoted as ${\upeta }_{\mathrm{i}}$ and ${\upepsilon }_{\mathrm{ij}}$, respectively. Here, subscripts i and j denote the earthquake event and the station number, respectively. These residual terms are assumed to be independent and follow a zero-mean normal distribution. The between-event and within-event residual component has a standard deviation of τ and σ, respectively. Recently, the mixed effect algorithm initially proposed by Abrahamson and Youngs (1992) has been modified by Khosravikia and Clayton (2021) and Mohammadi et al. (2023) regarding the likelihood function. In this study, the mixed-effect model is constructed following the method outlined by Khosravikia and Clayton (2021) and Mohammadi et al. (2023), where the model undergoes iterative training, using the ANN algorithm until meeting a convergence criterion. For this purpose, convergence is met when the relative difference between two consecutive log-likelihood values falls below 0.001. Given a GMM, the total standard deviation (Φ) is calculated as the square root of the sum of squares of the between-event (τ) and within-event (σ) standard deviations, expressed as:

7 $$\upphi =\sqrt{{\uptau }^{2}+{\upsigma }^{2}}$$

ANNs are sophisticated systems of interconnected neural units that can learn from input data and adapt their behavior accordingly. ANNs function in two main phases: learning and recall. ANNs operate in two primary stages: training and inference. In the training stage, the network learns by adjusting the weights of connections between input and output layers using known datasets. In the inference stage, the network applies these learned weights to new input data to make predictions. Various studies have demonstrated the efficacy of ANN-based GMMs in capturing intricate patterns in seismic data (Dhanya and Raghukanth 2018; Karimzadeh et al. 2023a; Mohammadi et al. 2023).

This study employs the Scikit-learn Multi-layer Perceptron Regressor (Pedregosa et al. 2011), a well-established Python package. The grid search method, combined with cross-validation, is used to identify the best settings for crucial parameters in the model, including the number of hidden layers, the number of nodes per layer, and the activation function. To develop the ANN-based GMM, we focused on strong motions and excluded synthetics from distant stations. This is achieved using a Z-score statistical technique, which restricted the dataset to R_JB_ values between 4 and 54 km, resulting in a refined total of 199,531 motions for training and testing. The dataset is divided into 80 percent for training the model and 20 percent for model validation. The optimal setting involves a one-layer neural network architecture, including an input layer, a hidden layer with 5 neurons using a log-sigmoid activation function, and an output layer.

**Fig. 12:**
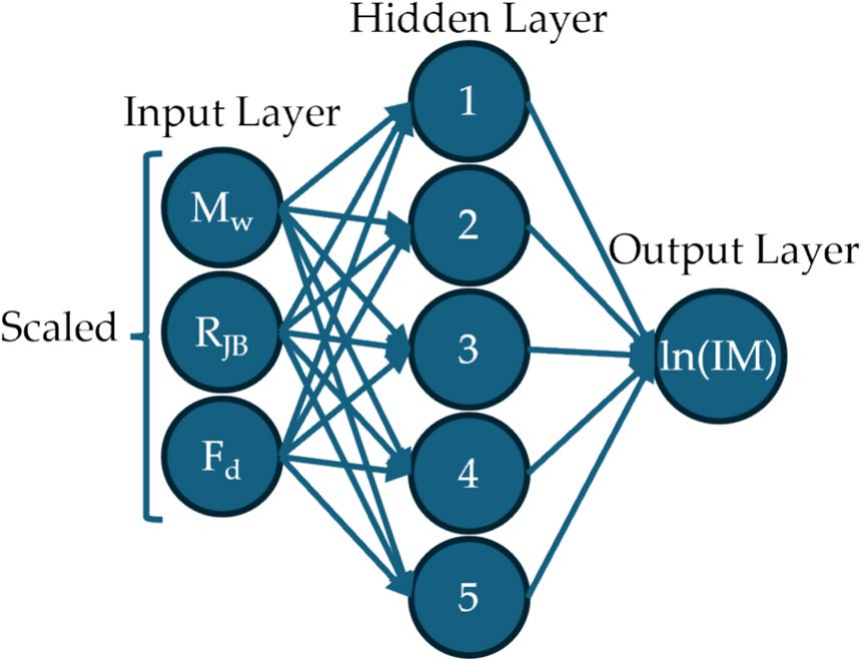
Schematic representation of the ANN model

Herein, the input parameters are M_w_, R_JB_, and F_d_, which are preprocessed using a scaler that removes the median and scales the data according to the quantile range to ensure normalized input distributions. The output layer consists of the prediction of ground motion IMs in logarithmic scale, specifically ln(PGA), ln(PGV), and ln(PSA) in the period range of 0.05–2.0 s. The Marquardt–Levenberg algorithm (Marquardt 1963) is used to minimize the error between the target values and the actual output values in the model. Figure 12 illustrates a schematic representation of the employed ANN algorithm.

## ANN-based ground motion model

### Performance of the ANN-based GMM

**Fig. 13:**
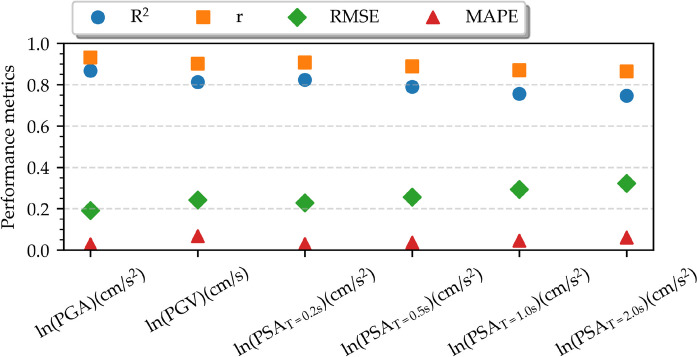
Performance metrics in terms of R^2^, r, RMSE, and MAPE for selected IMs

The performance of the ANN-based GMM is evaluated using test data, using a range of statistical metrics. Specifically, the selection of the coefficient of determination (R^2^), Pearson correlation coefficient (r), root-mean-square error (RMSE), and mean absolute percentage error (MAPE) provides valuable insights into the fit, correlation, accuracy, and relative error of the model, respectively. Figure 13 illustrates the evaluated metrics for the proposed GMM across selected IMs. The selected IMs, namely ln(PGA), ln(PGV), and ln(PSA) at periods of 0.2 s, 0.5 s, 1 s, and 2 s, are chosen as representatives of a wide range of frequencies of interest. The performance metrics, encompassing RMSE, R^2^, r, and MAPE, demonstrate uniformity across all IMs, indicating consistent performance without significant deviation for any specific IM. The mean RMSE value is around 0.25, showing a slight increase towards ln(PSA) at longer periods. Moreover, an opposite trend is observed in the R^2^ and r metrics for ln(PSA) as the periods increase, indicating a slight decline. Nonetheless, the mean R^2^ value remains close to 0.82, indicating a close match between the predicted and real values. With a mean r value of approximately 0.90, there is strong evidence of a linear correlation between the predicted and real values. The MAPE and RMSE values across all IMs exhibit a narrow range, averaging approximately 0.05 and 0.25, respectively. Expected differences between MAPE and RMSE arise mostly due to their distinct error assessments. MAPE shows the relative absolute magnitude of errors, while RMSE represents the overall squared magnitude. Consequently, RMSE is further sensitive to the data scaling and outliers within the model. The model exhibits robust performance when applied to unseen data, as confirmed by the overall consistency of the evaluated metrics using the test dataset.

**Fig. 14:**
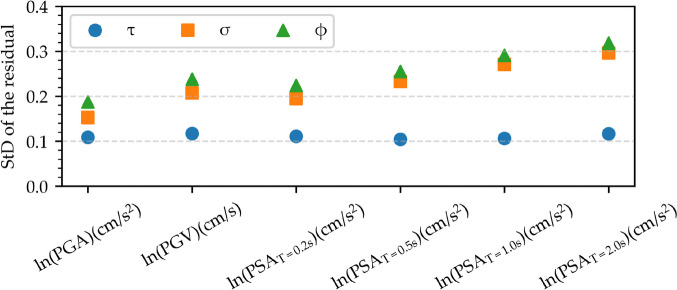
Distribution of between-event (τ) and within-event (σ) uncertainties, along with total uncertainties (ϕ), for ln(PGA), ln(PGV), and ln(PSA) at periods ranging from 0.05 s to 2 s

Subsequently, the model's bias regarding M_w_ and R_JB_ is examined by analyzing the residuals. This assessment partitions the total uncertainty into between-event (τ) and within-event (σ) uncertainties, representing the standard deviation of residuals, respectively, for the earthquake source and source-to-site characteristics. Figure 14 illustrates the distribution of between-event, within-event, and total uncertainties for ln(PGA), ln(PGV), and ln(PSA) at periods of 0.2 s, 0.5 s, 1 s, and 2 s. Across all IMs, the between-event residual consistently remains smaller than the within-event residual. Longer periods exhibit a tendency for the within-event residual of ln(PSA) to escalate, leading to an increase in total uncertainty.

**Fig. 15:**
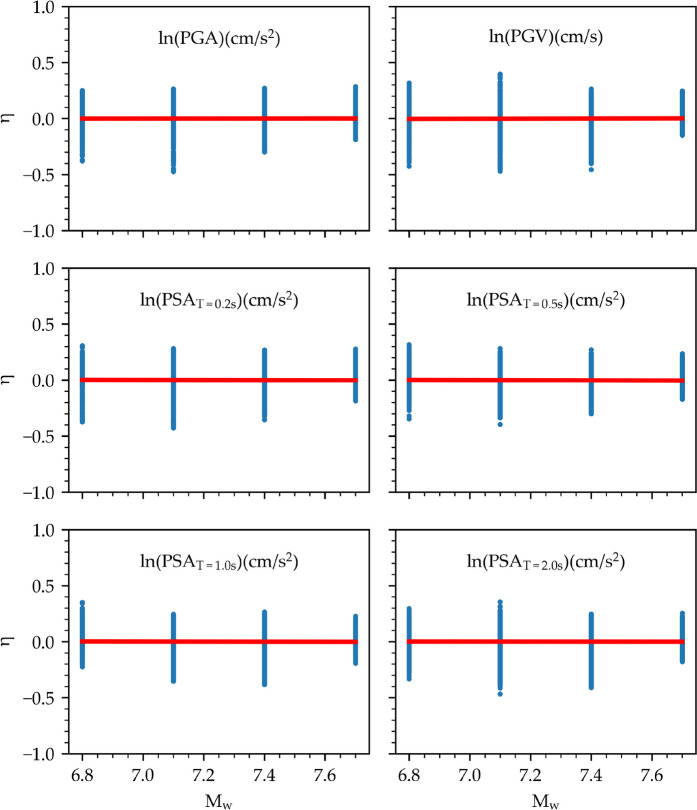
Distribution of the between-event residual (η) with respect to M_w_ for ln(PGA), ln(PGV), and ln(PSA) at periods of 0.2 s, 0.5 s, 1.0 s, and 2.0 s

**Fig. 16:**
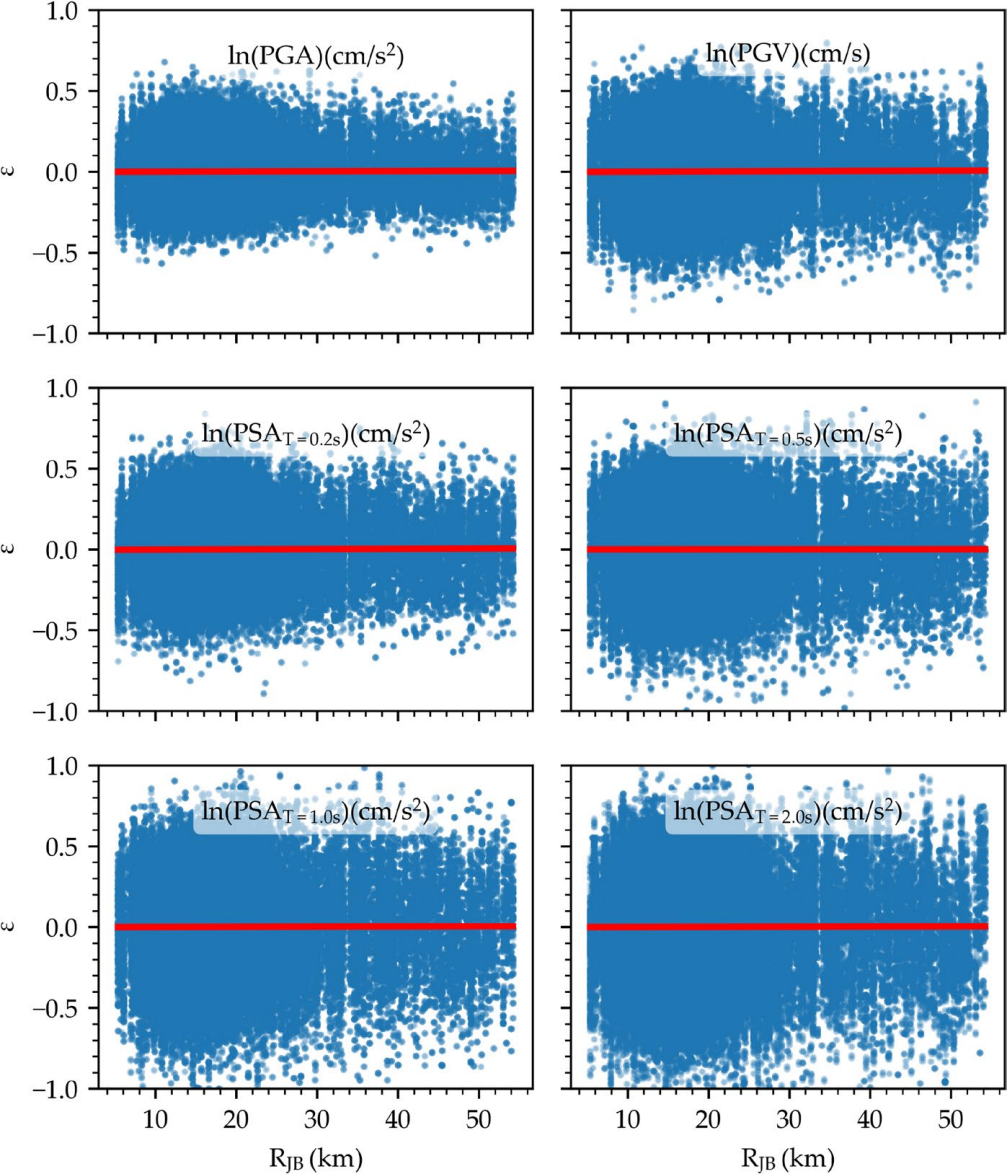
Distribution of the within-event residual (ϵ) with respect to R_JB_ for ln(PGA), ln(PGV), and ln(PSA) at periods of 0.2 s, 0.5 s, 1.0 s, and 2.0 s

A further residual analysis is performed for the considered IMs. Figures 15 and 16 illustrate the distribution of between-event and within-event residuals with respect to M_w_ and R_JB_ for selected IMs, respectively. The between-event residuals fluctuate within the range of –0.4 to 0.4, whereas the within-event residuals exhibit a wider span between –1.0 and 1.0, consistent with findings from previous studies (Akkar et al. 2014; Kale et al. 2015; Karimzadeh et al. 2023a; Mohammadi et al. 2023). It is important to note that the model's predictions are most reliable within the training data range (6.8 ≤ M_w_ ≤ 7.7, 4 km ≤ R_JB_ ≤ 54 km) and can be less accurate when applied outside of that range.

The red lines in Figs. 15 and 16 represent the mean of residuals with respect to the independent variables. The absence of discernible trends in the mean residuals for both between-event and within-event categories across all IMs indicates the unbiased nature of model errors. The extremely narrow confidence intervals, rendering them invisible on the plot, add further weight to this observation. Therefore, the model shows no systematic bias towards M_w_ and R_JB_.

### Validation of the ANN-based GMM

**Fig. 17:**
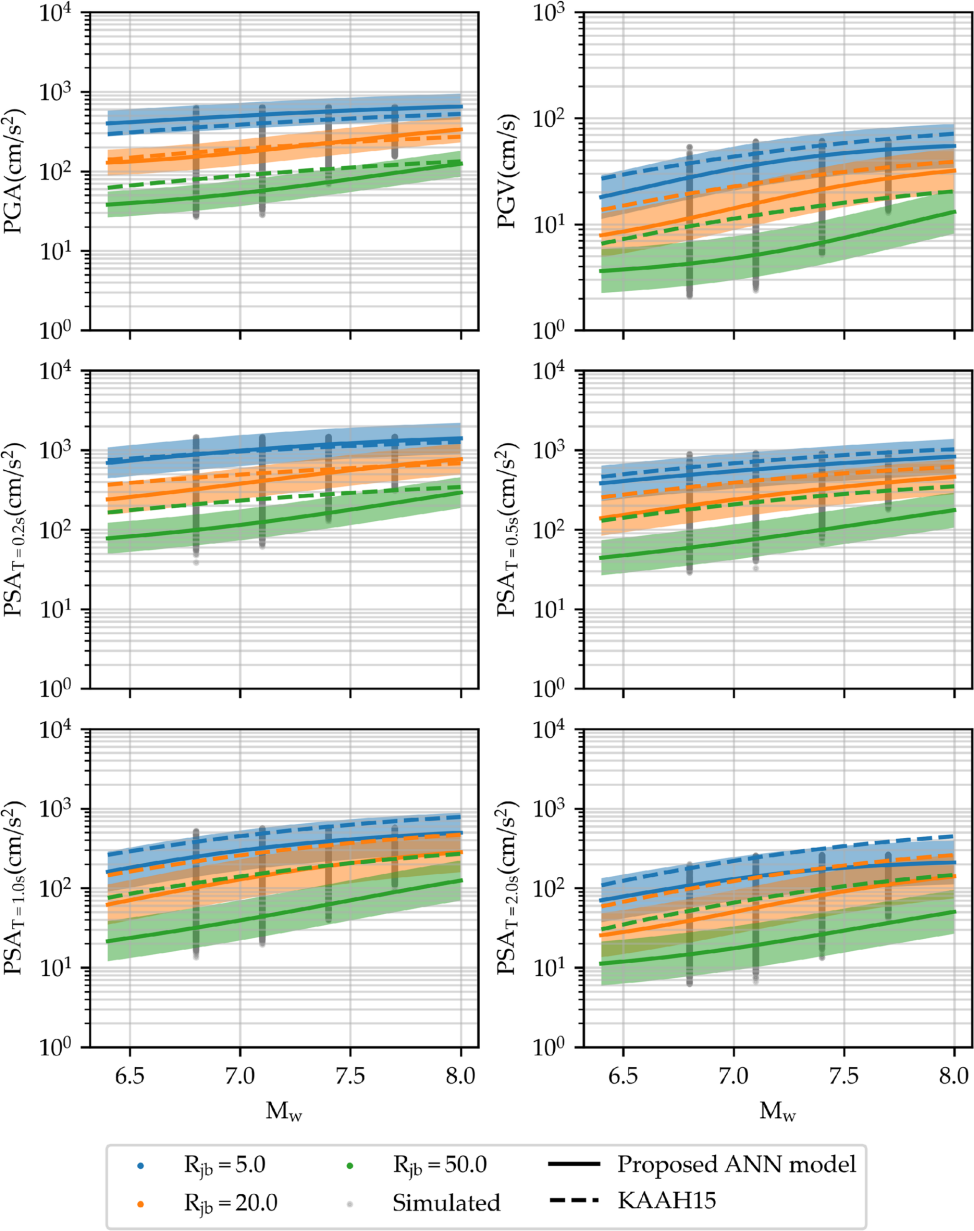
Variation of PGA, PGV, and PSA at periods of 0.2, 0.5, 1.0, and 2.0 s with respect to M_w_ for a strike-slip fault mechanism, V_s30_ = 310 m/s, and R_JB_ values of 5.0, 20.0, and 50.0 km. The proposed model is shown within two standard deviations

**Fig. 18:**
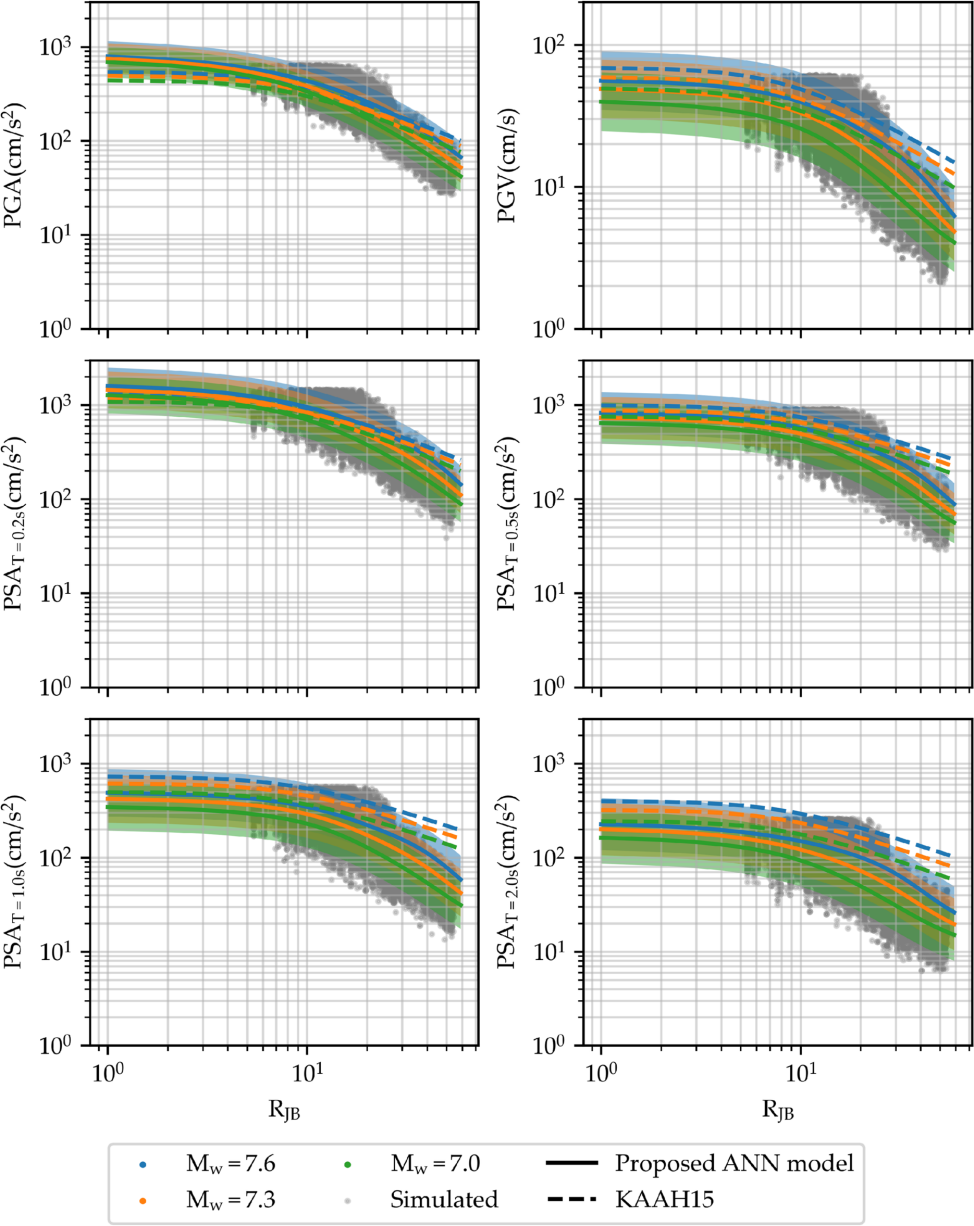
Variation of PGA, PGV, and PSA at periods of 0.2, 0.5, 1, and 2 s with respect to R_JB_ for a strike-slip fault mechanism, V_s30_ = 310 m/s, and M_w_ values of 7.0, 7.3, and 7.6 km. The proposed model is shown within two standard deviations

The model adequacy is attained by evaluating the attenuation pattern of IMs across a range of M_w_ and R_JB_ values with a Vs30 of 310 m/s, a strike-slip mechanism, and a mean F_d_ of 10 km. Figure 17 illustrates the variation of selected IMs with respect to M_w_ for different R_JB_ values of 5.0, 20.0, and 50.0 km. IMs acquired from simulated earthquake events are represented by filled gray dots. Additionally, a comparison with KAAH15, a parametric GMM tailored to Iran, is presented for further analysis. Results indicate that higher M_w_ and lower R_JB_ values correlate with elevated levels of all selected IMs. Similarly, Fig. 18 demonstrates the variation of these IMs with R_JB_, for different M_w_ values of 7.0, 7.3, and 7.6, alongside a comparison to the KAAH15 GMM. It is observed that as R_JB_ increases, there is a consistent decrease in PGA, PGV, and PSA across all periods, a trend effectively captured by the proposed GMM, highlighting its ability to reflect distance-dependent attenuation. Moreover, the relationship between higher M_w_ and amplified IMs remains consistent with prior observations, as the magnitude increase correlates with a greater energy release during seismic events (Karimzadeh et al. 2023a). The analysis reveals a strong alignment between the proposed ANN-based GMM using simulated data and the empirical GMM derived from real data, notably in PGA and PSA at the period of 0.2 s. Nonetheless, discrepancies become more pronounced for other IMs, particularly noticeable in PSA at longer periods, where the disparity increases. Some of these discrepancies might be attributed to the selection of the model parameters in the simulations as well as the potential limitations of the stochastic finite-fault approach in accurately capturing the longer period content of the records and basin effects.

## Conclusions

This study employs stochastic finite-fault ground motion simulation based on the dynamic corner frequency to simulate alternative scenario events at different magnitude values within the city center of Tabriz, Iran. The effects of diverse hypocenter locations and rupturing fault planes for specific magnitudes are evaluated statistically in detail. The analyses focused on the city center, allowing for the assessment of variations in ground motion intensity resulting from different hypocenter and fault plane configurations.

The results are validated against empirical ground-motion models (GMMs), namely BA08 (Boore and Atkinson 2008), AC10 (Akkar and Cagnan 2010), ASB14 (Akkar et al. 2014), and KAAH15 (Kale et al. 2015). The findings reveal the validity of the simulations from a seismological point of view, exhibiting considerable variability in ground motion intensity measures (IMs) across each site of interest. This variability is attributed to uncertainties in the earthquake hypocenter and the fault plane rupture. Moreover, the evaluation of the inter-period correlation of the residual pseudo-spectral acceleration (PSA) serves as an additional step in the validation process. The highest values of all ground motion IMs are consistently observed in the immediate vicinity of the North Tabriz Fault (NTF) line, underscoring the significance of this area in terms of potential hazards.

The findings suggest that higher earthquake magnitudes result in higher values of seismic IMs such as peak ground acceleration (PGA), peak ground velocity (PGV), and PSA across different periods. Specifically, comparing the moment magnitude (M_w_) of 7.7 with that of 6.8, 7.1, and 7.4 reveals a significant reduction of approximately 65%, 45%, and 15%, respectively, based on the mean average of the IMs. Furthermore, the considerable standard deviations concerning the mean values across various M_w_ and IMs indicate a broad spectrum of potential ground-shaking levels. The varying seismic intensity experienced during earthquakes of similar magnitudes within the region underscores the significant hazard faced by the city center, especially for the highest M_w_ of 7.7, stressing the importance of preparedness. Estimations of Modified Mercalli intensity (MMI) values across various scenarios further highlight the increased vulnerability of Tabriz city center to significant potential hazards.

A local ground motion model (GMM) is developed by training artificial neural networks (ANN) on simulated ground motions. The developed ANN-based GMM incorporates moment magnitude (M_w_), Joyner-Boore distance (R_JB_), and focal depth (F_d_) as input parameters, enabling the estimation of ln(PGA), ln(PGV), and ln(PSA) at various periods. Through residual analysis, the uncertainty of the GMM is quantified in terms of between-event and within-event variations. Comparison with an empirical GMM tailored to Iran reveals the robust performance of the proposed GMM in accurately capturing observed patterns in ground motions despite some discrepancies for spectral ordinates at longer periods. This might be attributed to the potential limitations of the stochastic finite-fault approach in accurately capturing the longer period content of the records and basin effects. Moreover, an online platform is offered to ensure a user-friendly application of the developed GMM for end-users.

The analyses in this study are performed with uniform soil conditions. Future studies should aim to address the uncertainty stemming from input parameters, particularly site parameters along with the simulation methodology further to enhance our understanding of seismic hazards in the region. Additionally, future studies could explore the integration of these findings with vulnerability and exposure data to develop a comprehensive risk assessment framework.

## Data Availability

This study uses the Streamlit package in Python to construct a user-friendly graphical interface tool, facilitating convenient access to the ANN-based ground motion model (GMM). The code can be found at https://github.com/S-M-S–H/Tabriz-GMM-ANN, while the interface tool itself is accessible via https://tabriz-gmm-ann.streamlit.app/. Users are prompted to input the parameters of a scenario earthquake, such as moment magnitude (Mw), Joyner-Boore distance (RJB), and focal depth (Fd). The software then generates outcomes in terms of intensity measures (IMs), encompassing peak ground acceleration (PGA), peak ground velocity (PGV), and pseudo spectral acceleration (PSA). Finally, all data supporting this paper will be made available upon request to the corresponding author.
